# SUICIDAL IDEATION AMONG OLDER ADULTS LIVING WITH CHRONIC ILLNESS

**DOI:** 10.1093/geroni/igac059.1888

**Published:** 2022-12-20

**Authors:** Abigail Poe, Frank Puga

**Affiliations:** The University of Alabama at Birmingham, Birmingham, Alabama, United States; The University of Alabama at Birmingham, Birmingham, Alabama, United States

## Abstract

Older adults living with chronic illnesses, such as diabetes, cancer and heart disease, have an increased risk of poor mental health outcomes. While a link between living with chronic illness and depression have been examined in previous studies, relatively little is known about factors that increase and decrease the risk of suicidal ideation among older adults living with chronic illness. Using data from the third wave of the Midlife in the United States (MIDUS) database, we examined the relationship between living with a chronic illness were more likely to endorse thinking about death in the previous two weeks ( OR=3.17, CI: 1.16 – 8.65, p=0.24). The analysis also revealed that the likelihood of suicidal ideation in the previous two weeks increased with the number of chronic conditions reported by participants (OR=1.128, CI: 1.04 – 1.23, p=0.005). The results of this study are consistent with previous studies suggesting a relationship between older adults living with a chronic condition are at a higher risk of suicidal ideation. Findings from this study can help inform intervention development to support mental health of older adults living with chronic illness. Future studies are needed to examine additional psychosocial factors that may mediate the relationship between living with a chronic illness and suicidal ideation among older adults.

